# Chloral Anæsthesia in Children

**Published:** 1878-12

**Authors:** 


Chloral Anæsthesia in Children.—The following is borrowed from the *Paris Medical* of August 26th: M. Bouchut has administered chloral to more than ten thousand patients without having to register a single untoward effect. The physician to the Hospital for Children has arrived at the following conclusions, which may serve to guide physicians in practice:

The hydrate of chloral is better borne by children than in adults.

Chloral may be administered to children a long time without danger. (Some children affected with chorea have taken as high as 100 or 120 grammes of chloral in a month.)

According to the age, 1, 2 or 3 grammes and more administered by the stomach produce a complete anæsthesia which lasts from three to six hours. Under seven years the dose should not exceed three grammes, and between 2 and 5 years two gr. should be the limit.

It is given at one dose dissolved in 100 gr. of syrup.

Given by injection or suppository it produces the same effect as in the stomach.

This anæsthesia which can be produced so easily in children with chloral (complete one hour after administration), can be utilized in extracting teeth, opening abscesses, breaking up ankyloses, using caustics, thoracentesis and a certain number of surgical operations. —*Lyon Medical,* Sept. 1, 1878.—*Cincinnati Lancet.* R. B. D.

